# The burden and trends of headache disorders among the population aged 15–39: a study from 1990 to 2019

**DOI:** 10.1186/s10194-023-01703-0

**Published:** 2023-12-13

**Authors:** Ruixia Yuan, Zhuang Tong, Guoliang Xiang, Yingying Xie, Kaixiang Li, Liang Zhang, Xueqing Wang

**Affiliations:** 1 Clinical Big Data Center, The First Affiliated Hospital of Zhengzhou University, Zhengzhou, Henan Province China; 2 Henan Academy of Medical Big Data, Zhengzhou, China; 3 Department of Neurology, The First Affiliated Hospital of Zhengzhou University, Zhengzhou, Henan Province China; 4 Department of Scientific Management, The First Affiliated Hospital of Zhengzhou University, Zhengzhou, Henan Province China; 5 Department of Cardiovascular Surgery, The First Affiliated Hospital of Zhengzhou University, Zhengzhou, Henan Province China

**Keywords:** Headache disorders, Adolescent and young adults, Disease burden, Temporal trend

## Abstract

**Background:**

To analyze the global burden of headache disorders in adolescents and young adults (AYAs).

**Methods:**

Data of headache disorders in the 15–39 age groups were extracted from GBD 2019. The age-standardized rates (ASRs) of incidence, prevalence, and years lived with disability (YLDs) rate were used to describe the burden. Estimated Annual Percentage Changes (EAPCs) were used to describe the trend from 1990 to 2019.

**Results:**

In 2019, the age-standardized incidence rate (ASIR), age-standardized prevalence rate (ASPR), and age-standardized YLDs rate (ASYR) in AYAs were 42,473.18 (95% CI: 34,836.35, 50,858.30) per 100,000, 12,566.18 (95% CI: 8542.10, 16,915.68) per 100,000 and 790.32 (95% CI: 129.56, 1786.52) per 100,000, respectively. From 1990 to 2019, the ASRs showed increasing trends, with EAPCs of 0.04 (95% CI: 0.03, 0.06), 0.05 (95% CI: 0.03, 0.07), and 0.08 (95% CI: 0.07, 0.10). Migraine accounted for 91.54% of headache-related YLDs. The burdens in females were higher than those in males, especially for ASYR. This burden was greatest in the high sociodemographic index (SDI) super region. In the temporal trend of ASIR, 127 (62.25%) countries showed upward trends, mainly distributed in East Asia, Central Asia, West Africa, and Western Latin America.

**Conclusions:**

The burden of headache disorders in the global population aged 15–39 is severe, especially among females and in countries with high SDI. Furthermore, this burden has been steadily increasing over the past three decades. Those findings assist in implementing targeted intervention measures.

**Supplementary Information:**

The online version contains supplementary material available at 10.1186/s10194-023-01703-0.

## Introduction

Headache is one of the most common neurological disorders [1, 2]. Headaches can occur in all age groups, with a particularly heavy burden on young and middle-aged adults [3]. Adolescents and young adults (AYAs) constitute a subpopulation situated between children and middle-aged or elderly individuals [4]. The age group of 15 to 39 years is widely recognized as the age range defining AYAs, comprising approximately 38% of the global population [5]. They are the primary focus of education, employment, and entrepreneurship initiatives, acting as catalysts for innovation and change. Governments and organizations frequently allocate substantial resources to support and foster the development of this demographic [6]. However, in contrast to children and older adults, the disease burden and its risk factors specific to AYAs have not received more special attention in meeting age-related treatment and care needs. Headache is an important cause of physical activity restriction, lack of social activity, and decreased academic and work productivity in AYAs [7]. Additionally, headaches are associated with comorbidities such as allergies, sleep disorders, and psychiatric comorbidities [8]. This undoubtedly exacerbates emotional and behavioral issues in adolescents and young adults.

As early as 2004, the World Health Organization's European office took the lead in launching "Lifting the Burden" to actively promote fair and effective prevention and treatment of headaches worldwide [9]. Existing studies on the burden of headache disorders have focused on populations of all ages, specific regions, or specific types of headaches [1, 3, 10]. To our knowledge, a comprehensive assessment of the burden of headaches in the 15–39 age groups is largely unknown or unreported. We selected 39 as a convenient cutoff point for our age group of interest. In the last iteration of the Global Burden of Disease (GBD) study, GBD 2019 provided complete data on population mortality rates, 369 diseases and injuries, and 87 risk factors for the years 1990 to 2019 for the global, super regions, and 204 countries [11]. Therefore, it presents an excellent opportunity for the systematic analysis of the burden of headaches in this age group globally and in different regions.

The aim of this study was to analyze and report the burden of headaches in the 15–39 age groups, comparing the differences in its distribution and changes among different genders and regions with varying levels of economic development.

## Methods

### Data sources

GBD 2019 is a multinational collaborative study that aimed to estimate the burden of disease worldwide, measuring the epidemiological levels and trends of infectious diseases, non-communicable diseases, and injuries [12]. The foundational data for the GBD study is derived from sources such as population censuses, household surveys, civil registrations, and population vital statistics. It also includes disease registries, disease notifications, healthcare service utilization, air pollution monitoring devices, satellite imaging, and more [5]. Through systematic synthesis and standardization, the GBD 2019 project estimate the burden associated with diseases and injuries. Additional details on the calculation methods have been disclosed in previous publications of GBD 2019 [5, 11].

According to the research purpose, we set the parameters in the free data download interface provided by GBD 2019.For example, under "Cause," we selected "headache disorders," "Migraine," and "TTH"; under "Measure," we chose "incidence," "prevalence," and "YLDs"; under "Age," we selected "15–39 years," "15–19 years," "20–24 years," "25–29 years," "30–34 years," and "35–39 years"; and under "Sex," we selected "both," "male," and "female," and so on.

### Case definition

According to the GBD 2019 migraine is defined as a disabling primary headache disorder characterized by recurrent episodes of moderate to severe unilateral throbbing pain. When a patient's symptoms meet all five major diagnostic criteria proposed by the International Classification of Headache Disorders (ICHD-3), it is classified as a definite migraine [3]. Tension-type headache (TTH) is defined as a mild to moderate diffuse, non-throbbing, pressing, bilateral etc. in the head and neck region. The diagnostic process for TTH is essentially the same as that for migraine. If a patient's symptoms meet all five major diagnostic criteria proposed by ICHD-3, it is defined as TTH [13]. In this study, the coding for migraine and TTH in the International Classification of Diseases 10th Revision (ICD-10) is as follows: G43-G43.919, G44.2-G44.229, and G44.4-G44.41.

### Estimation of headache burden in GBD 2019

The GBD 2019 employed the Bayesian meta-regression modeling tool DisMod-MR 2.1 to ensure consistency among these burden outcome indicators. Uncertainty intervals were provided for each point estimate using the 25th and 75th sorted values of the posterior distribution, resulting in1000 draws [11, 14]. The calculation method for prevalence is the number of cases (n) multiplied by 100,000 divided by the population, while the incidence is the number of new cases (n) multiplied by 100,000 divided by the population [15]. There were no direct mortality estimates associated with headache disorders, and the YLDs metric represents Disability-Adjusted Life Years (DALYs) [16].

### Statistical analysis

According to the world standard population (WHO 2000–2025), we calculated the age-standardized rates (ASRs) [17]. The ASRS are calculated using the following equation: ${\text{Age}}-\text{standardized rate } = \frac{{\sum }_{i=1}^{A}{a}_{i}{w}_{i}}{{\sum }_{i=1}^{A}{w}_{i}}$. Where a_i_ represents the age-specific rate and w_i_ is the weight in the same age subgroup of the chosen reference standard population. We described and compared the burden of headache disorders in AYAs using age-standardized incidence rate (ASIR), age-standardized prevalence rate (ASPR), and age-standardized YLDs rate (ASYR) with 95% uncertainty intervals (UIs).

The estimated annual percentage changes (EAPCs) in ASRs were used to assess the trend in the burden. A regression line model ln (ASR) = α + βx + ɛ, was fitted to the natural logarithm of the ASRs, where x represents the calendar year [18]. The EAPC and its 95% confidence interval (CI) were calculated using the following regression model: y = 100 × (exp (β)-1), with y denoting the EAPC. If the lower limit of the 95% CI for the EAPC estimate was greater than 0, an increasing trend in ASRs during the observation period was considered. Conversely, if the upper limit of the 95% CI of the EAPC estimate was less than 0, a decreasing trend was recognized. When the 95% CI included 0, the trend change was not statistically significant [19].

We examined the differences in this burden among different sexes, age groups (15–19, 20–24, 25–29, 30–34, and 35–39 years), and sociodemographic index (SDI, five categories). The smoothing splines models and Pearson correlation were used to describe and assess the correlation of countries' SDI and the burden of headache disorders in AYAs.

Data collation, analysis, and graphical rendering were performed using Python and R software (version 3.5.3).

## Results

### The global burden of headache disorders in AYAs

**Fig. 1:**
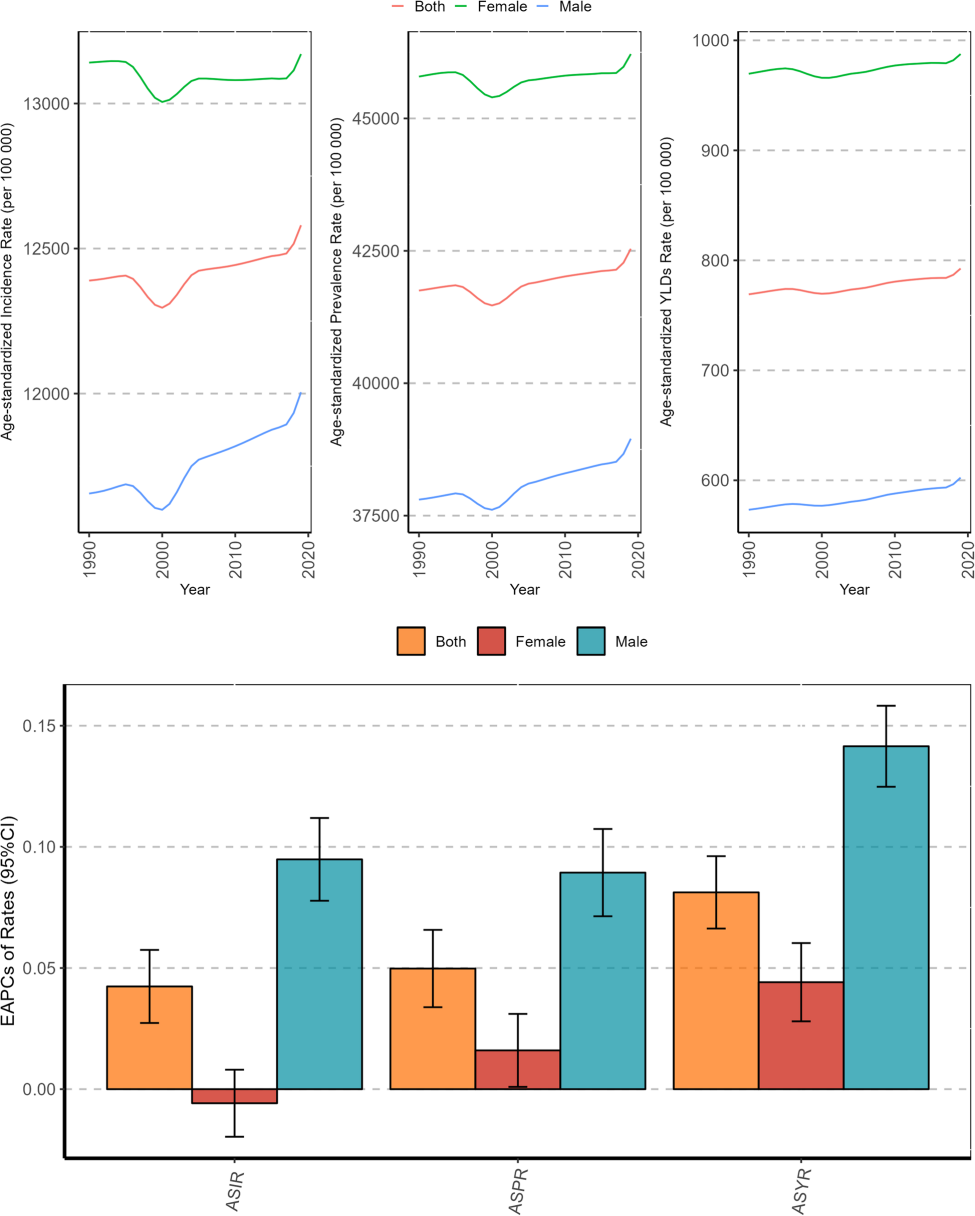
Temporal trend of headache disorders burden in adolescent and young adults by sex from 1990 to 2019

In 2019, there were 1262.47 (95% UI: 1131.42–1401.96) millions headache patients amongst global AYAs, with an ASPR of 42,473.18 (95% CI: 34,836.35, 50,858.30) per 100,000. There were 373.37 (95%UI: 311.66–226.34) millions of new headache patients, and the ASIR was 12,566.18 (95%CI: 8542.10, 16,915.68) per 100 000. YLDs caused by headache were 23.52 (95%UI: 3.88–52.40) millions, ASYR was 790.32 (95%CI: 129.56, 1786.52) per 100 000.In terms of changing trend, the burden of headaches in AYAs worldwide showed an increasing trend from 1990 to 2019. EAPCs of ASIR, ASPR and ASYR were 0.04 (95%CI: 0.03, 0.06), 0.05(95%CI: 0.03, 0.07) and 0.08(95%CI: 0.07, 0.10), respectively (Table 1, Fig. 1).

**Table 1:** The global burden of headache disorders in adolescent and young adults in 2019

Characteristics	Incidence, millions (95% UI)	Age-standardized incidence rate per 100 000 (95% CI)	Prevalence, millions (95% UI)	Age-standardized prevalence rate per 100 000 (95% CI)	YLDs, millions (95% UI)	Age-standardized YLDs rate per 100 000 (95% CI)
Global	373.37(311.66–439.49)	12,566.18(8542.10,16,915.68)	1262.47(1131.42–1401.96)	42,473.18(34,836.35,50,858.30)	23.52(3.88–52.40)	790.32(129.56,1786.52)
Gender						
Female	192.98(161.85–226.34)	13,156.78(9041.93,17,651.94)	677.16(608.92–746.60)	46,128.79(38,455.69,54,311.69)	14.47(2.28–32.90)	984.09(153.51,2261.73)
Male	180.39(149.70–213.73)	11,991.57(8055.68,16,228.17)	585.32(519.56–655.21)	38,907.33(31,216.08,47,509.86)	9.05(1.62–20.18)	601.19(106.34,1346.15)
Types						
Migraine	43.79(35.59–53.52)	1477.65(939.64,2146.99)	581.76(488.31–696.29)	19,550.72(15,450.96,24,545.46)	21.53(2.54–49.94)	723.50(88.02,1715.78)
Tension-type headache	329.57(267.55–393.98)	11,088.53(7084.34,15,380.73)	964.81(809.58–1155.24)	32,458.46(22,393.93,44,328.62)	1.99(0.53–7.42)	66.82(16.17,249.39)
SDI						
Low SDI	53.02(44.19–63.10)	11,967.98(8031.97,16,216.67)	177.02(156.35–198.54)	40,007.49(32,260.07,48,736.29)	3.15(0.54–7.01)	714.38(123.30,1604.57)
Low-middle SDI	92.95(77.76–109.78)	12,670.08(8604.16,17,049.89)	313.28(279.51–348.26)	42,720.38(34,967.70,51,211.88)	5.75(0.85–13.03)	785.14(117.07,1795.19)
Middle SDI	112.15(93.86–131.93)	11,965.17(8148.58,16,117.05)	387.44(346.90–429.71)	41,310.15(33,934.27,49,441.17)	7.48(1.21–16.85)	795.02(127.37,1815.70)
High-middle SDI	64.43(53.35–75.97)	12,405.30(8455.75,16,698.31)	218.42(195.85–242.78)	41,925.98(34,381.84,50,091.27)	4.13(0.79–9.02)	784.57(143.72,1749.58)
High SDI	50.61(41.93–59.66)	15,249.86(10,405.08,20,545.35)	165.58(149.61–182.93)	49,596.13(41,304.62,58,418.40)	3.01(0.52–6.66)	894.48(144.14,2009.97)
Geographic Super- Region						
East Asia	49.88(41.28–59.10)	9527.02(6506.74,12,850.75)	175.08(156.29–195.69)	33,434.91(27,080.91,40,547.08)	3.39(0.59–7.60)	636.50(107.64,1436.61)
Southeast Asia	34.99(29.21–41.22)	12,864.37(8776.71,17,366.16)	121.20(108.47–134.76)	44,541.31(36,643.02,53,467.98)	2.40(0.31–5.70)	880.86(113.02,2099.98)
Oceania	0.62(0.51–0.73)	11,419.85(7593.47,15,623.22)	2.16(1.90–2.46)	39,955.82(31,626.74,49,471.03)	0.04(0.01–0.09)	751.93(105.47,1742.80)
Central Asia	5.68(4.67–6.72)	14,945.96(9988.53,20,370.35)	18.30(16.24–20.67)	48,102.80(38,412.62,58,428.80)	0.29(0.06–0.63)	754.17(141.59,1663.25)
Central Europe	5.32(4.38–6.26)	14,863.18(9973.21,20,103.97)	17.06(15.26–19.18)	47,448.98(38,419.71,57,185.57)	0.28(0.07–0.62)	771.12(171.97,1678.91)
Eastern Europe	11.19(9.13–13.21)	16,294.28(11,088.79,21,935.74)	35.39(31.81–39.38)	50,953.33(41,824.04,60,696.79)	0.60(0.17–1.25)	843.02(235.52,1764.10)
High-income Asia Pacific	7.73(6.38–9.21)	14,659.64(9835.73,19,869.19)	24.03(21.68–26.65)	45,230.49(37,057.85,54,049.84)	0.35(0.07–0.77)	650.87(131.19,1436.79)
Australasia	1.34(1.11–1.58)	13,728.66(9221.62,18,516.89)	4.35(3.88–4.87)	44,411.31(35,955.79,53,873.94)	0.08(0.01–0.17)	769.50(132.46,1717.45)
Western Europe	21.12(17.52–24.98)	16,079.66(10,902.72,21,756.14)	71.92(64.96–79.48)	54,418.79(45,523.54,63,819.15)	1.40(0.22–3.13)	1054.68(150.41,2419.53)
Southern Latin America	3.40(2.80–4.00)	13,340.38(8847.72,18,265.82)	10.79(9.52–12.17)	42,295.04(33,517.88,52,106.54)	0.17(0.04–0.38)	674.51(132.55,1482.03)
High-income North America	20.70(17.27–24.24)	17,011.68(11,677.43,22,794.27)	65.68(59.81–71.95)	53,792.38(45,278.23,62,684.99)	1.18(0.18–2.63)	962.31(139.82,2183.75)
Caribbean	2.12(1.75–2.51)	11,703.18(7650.28,16,044.93)	7.43(6.51–8.41)	40,954.52(32,535.80,50,502.62)	0.14(0.02–0.31)	751.40(118.11,1723.19)
Andean Latin America	2.50(2.07–2.99)	9726.59(6308.19,13,448.14)	8.78(7.64–10.07)	34,209.25(26,533.07,43,205.88)	0.15(0.03–0.32)	577.08(116.22,1270.98)
Central Latin America	12.28(10.22–14.52)	12,159.00(8122.05,16,435.12)	42.20(37.42–47.27)	41,794.35(33,912.65,50,700.52)	0.78(0.12–1.77)	773.22(121.38,1755.28)
Tropical Latin America	12.09(9.99–14.41)	13,559.23(9085.79,18,421.47)	43.60(39.48–48.05)	48,971.12(41,146.42,57,402.40)	0.89(0.11–2.06)	1003.67(115.80,2366.56)
North Africa and Middle East	29.92(24.78–35.43)	11,558.05(7736.24,15,673.34)	109.98(97.56–123.04)	42,462.83(34,576.81,51,238.46)	2.41(0.52–5.31)	927.35(196.45,2084.40)
South Asia	102.18(85.58–120.21)	13,311.59(9094.24,17,859.74)	337.85(302.37–373.66)	44,032.35(36,214.79,52,432.18)	6.04(0.81–13.61)	788.63(107.28,1810.84)
Central Sub-Saharan Africa	6.17(5.08–7.39)	12,064.71(8018.05,16,647.09)	20.62(17.98–23.45)	40,371.69(31,858.75,50,110.89)	0.36(0.07–0.79)	699.02(130.58,1545.76)
Eastern Sub-Saharan Africa	16.58(13.67–19.83)	10,080.31(6625.57,13,870.87)	53.80(46.72–61.30)	32,748.65(25,548.49,41,199.80)	0.85(0.20–1.85)	523.02(120.87,1133.06)
Southern Sub-Saharan Africa	4.28(3.56–5.07)	12,693.13(8629.01,17,170.48)	14.00(15.52–15.61)	41,479.30(33,708.25,50,146.55)	0.24(0.05–0.54)	723.28(138.78,1599.23)
Western Sub-Saharan Africa	23.27(19.26–27.55)	13,191.94(8891.95,17,884.13)	78.23(69.30–87.19)	44,382.99(36,244.33,53,270.15)	1.49(0.24–3.38)	846.77(138.71,1917.80)

### The burden of headache disorders in AYAs by super region

**Fig. 2:**
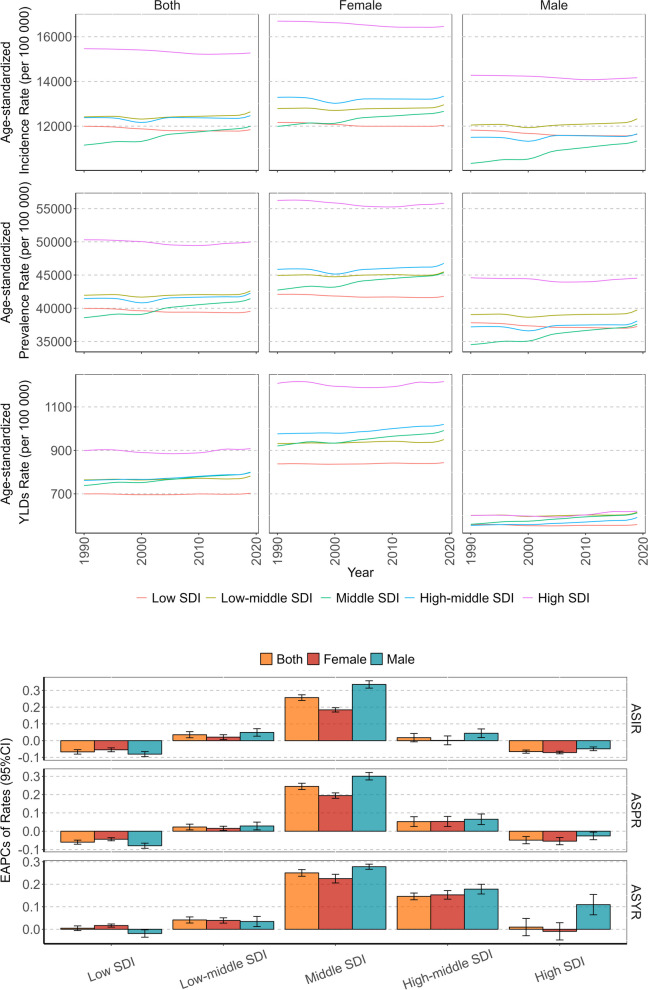
Temporal trend of headache disorders burden in adolescent and young adults by sex and SDI from 1990 to 2019

In five super-regions with different SDI levels, this burden was greatest in the high SDI region, with ASIR, ASPR and ASYR of 15,249.86 (95% CI: 10,405.08, 20,545.35) per 100 000, 49,596.13 (95% CI: 41,304.62, 58,418.40) per 100 000, and 894.48 (95% CI: 144.14, 2009.97) per 100 000, respectively. The lowest burden occurred in the low SDI and middle SDI regions. Among 21 geographic regions, AYAs in High-income North America and Western Europe had the highest burden, while the occurrence was relatively lower in East Asia. In terms of trends, the greatest increase in this burden in the last three decades was observed in the middle SDI region, with an EAPC of ASIR, ASPR, and ASYR of 0.26 (95% CI: 0.24, 0.27), 0.24 (95% CI: 0.23, 0.26), and 0.25(95% CI: 0.24, 0.27), respectively. Furthermore, this upward trend was more pronounced among males, with an EAPC of ASIR, ASPR, and ASYR of 0.26 (95% CI: 0.24, 0.27), 0.24 (95% CI: 0.23, 0.26), and 0.25(95% CI: 0.24, 0.27). The incidence and prevalence in low and high SDI regions showed negative trends. The EAPCs of ASIR and ASPR for the former were -0.07 (95% CI: **-**0.08, **-**0.05) and -0.06 (95% CI: **-**0.07, -0.05), respectively. For the latter, the EAPCs were -0.07 (95% CI: **-**0.08, **-**0.06) and -0.05 (95% CI: **-**0.07, **-**0.01), respectively (Table 1, Fig. 2).

### The burden of headache disorders in AYAs by country

**Fig. 3:**
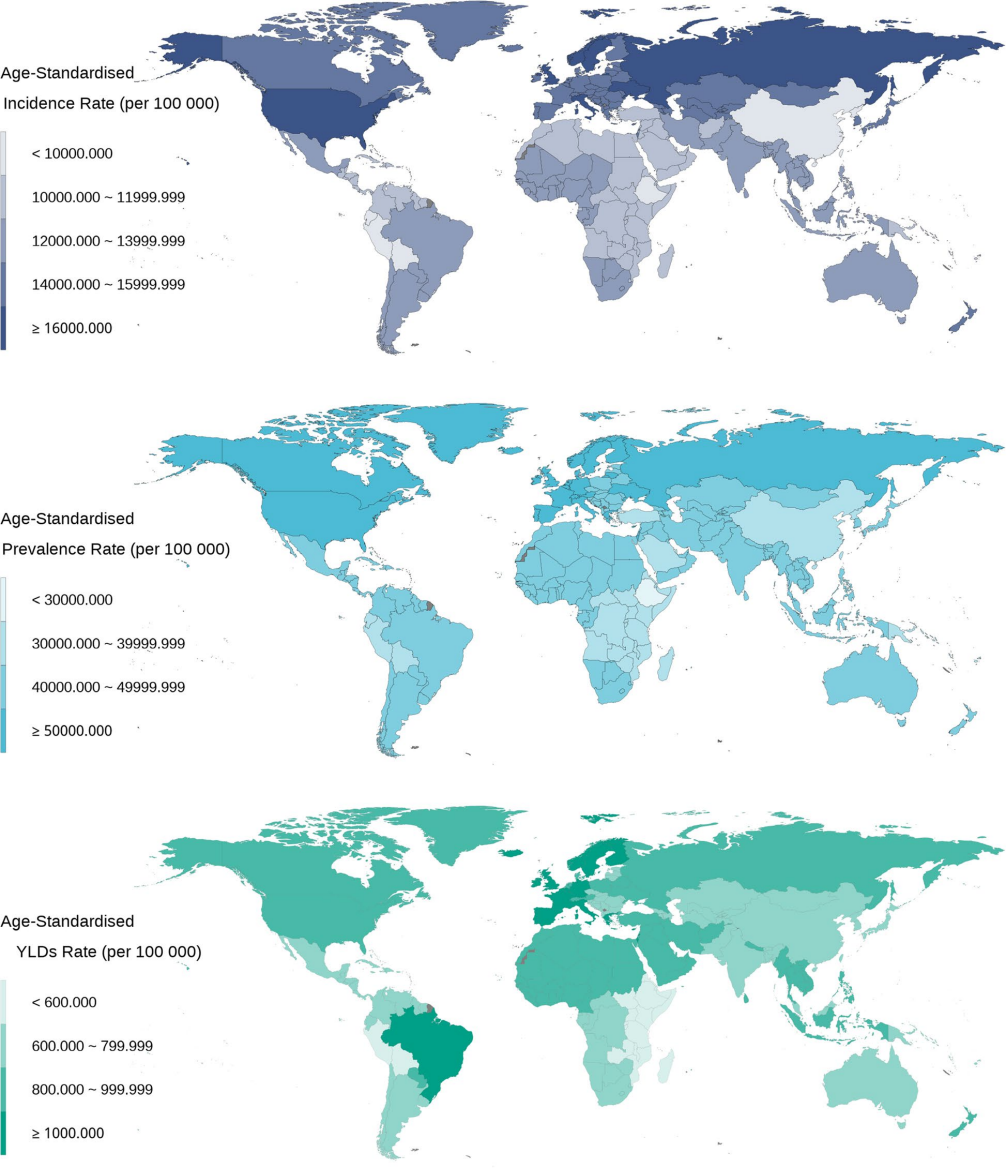
The distribution of headache disorders burden in adolescent and young adults by countries in 2019

Among all 204 countries, 40 (19.61%) countries had an ASIR exceeding 15,000 per 100,000. These countries were mainly concentrated in Eastern and Western Europe, Northern Asia, and North America. Italy had the highest ASIR, which was 17,462.52 (95% UI: 14,493.41–20,638.99)per 100 000.There were 7 countries with ASIR below 10,000 per 100,000, mainly concentrated in East Asia, Western Latin America, and East Africa. Ethiopia had the lowest ASIR, which was 8988.36 (7492.41, 10,785.75) per 100 000. Countries with ASYR exceeding 1000 per 100,000 were mainly distributed in Western Europe, North America, and Southeast Asia, while countries with ASYR lower than 600 per 100,000 were primarily found in East Africa and Western Latin America (Fig. 3).

**Fig. 4:**
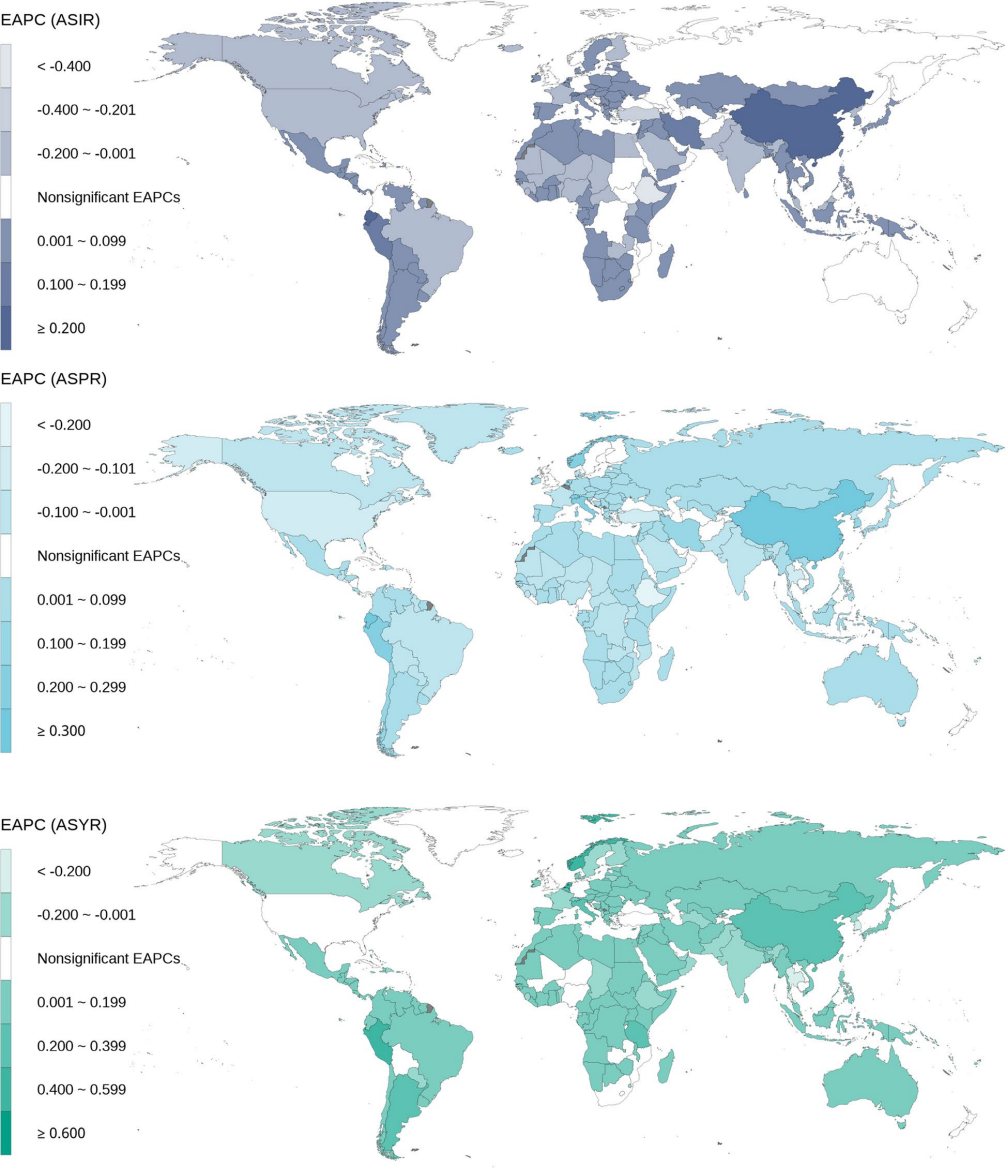
Temporal trend of headache disorders burden in adolescent and young adults by countries and regions from 1990 to 2019

In the temporal trend of ASIR from 1990 to 2019, 127 (62.25%) countries showed upward trends, mainly distributed in East Asia, Central Asia, West Africa, and Western Latin America. China, Ecuador, and Singapore were at the forefront of this upward trend, especially in China where the EAPC of ASIR was 0.36 (95% CI: 0.29, 0.43). 36(17.65%) countries showed downward trends, mainly distributed in North America, Western Asia, and East Africa. Ethiopia had the most prominent downward trend, with an EAPC of ASIR of -0.42 (95% CI: -0.50, -0.34). 41 (20.10%) countries had no statistically significant trend. The trend for prevalence rate showed a similar pattern and will not be further elaborated on here. In terms of ASYR, 133 (65.20%) countries showed upward trends, mainly concentrated in East Asia, Northern Europe, and Western Latin America. Singapore, Norway, Netherlands, and Peru exhibited the most significant trends, with all values exceeding 0.4 (Fig. 4).

### The global burden of headache disorders in AYAs by types

**Fig. 5:**
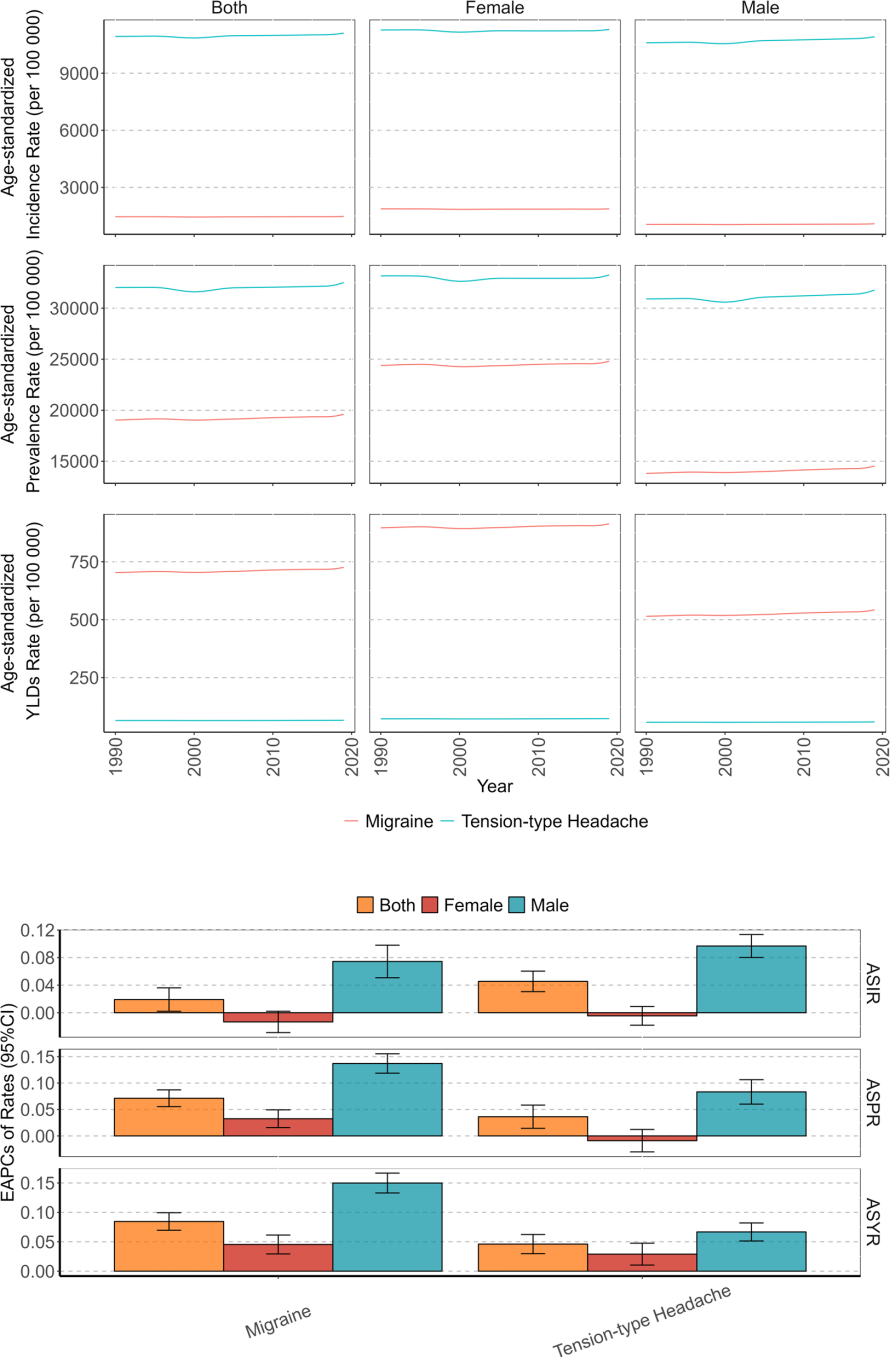
Temporal trend of headache disorders burden in adolescent and young adults by sex and types from 1990 to 2019

In 2019, TTH accounted for approximately 88.27% of new-onset headaches in AYAs, with a total of 329.57 (95% UI: 267.55–393.98) millions. Among the existing headaches in AYAs, TTH accounted for 76.42%, with a total of 964.81 (95% UI: 809.58–1155.24) millions. Migraine accounted for 91.54% of headache-related YLDs, totaling 21.53 (95% UI: 2.54–49.94) millions. The ASIR, ASPR, and ASYR of TTH were 11,088.53 (95% CI: 7084.34, 15,380.73) per 100,000, 32,458.46 (95% CI: 22,393.93, 44,328.62) per 100,000, and 66.82 (95% CI: 16.17, 249.39) per 100,000 respectively. The ASIR, ASPR, and ASYR of Migraine were 1477.65 (95% CI: 939.64, 2146.99) per 100,000, 19,550.72 (95% CI: 15,450.96, 24,545.46) per 100,000, and 723.50 (95% CI: 88.02, 1715.78) per 100,000, respectively. In terms of trend changes, TTH showed a more pronounced increase in incidence and prevalence rates, while Migraine displayed a more pronounced upward trend in YLDs rate. The EAPCs of ASIR, ASPR, and ASYR of TTH were 0.05 (95% CI: 0.03, 0.06), 0.04 (95% CI: 0.01, 0.05), and 0.05 (95% CI: 0.03, 0.06), respectively. The EAPC of ASIR, ASPR, and ASYR of Migraine were 0.02 (95% CI: 0.01, 0.04), 0.07 (95% CI: 0.06, 0.09), and 0.08 (95% CI: 0.07, 0.10), respectively (Table 1, Fig. 5, Figure S1, S2, S3).

### The global burden of headache disorders in AYAs by sex and age

**Fig. 6:**
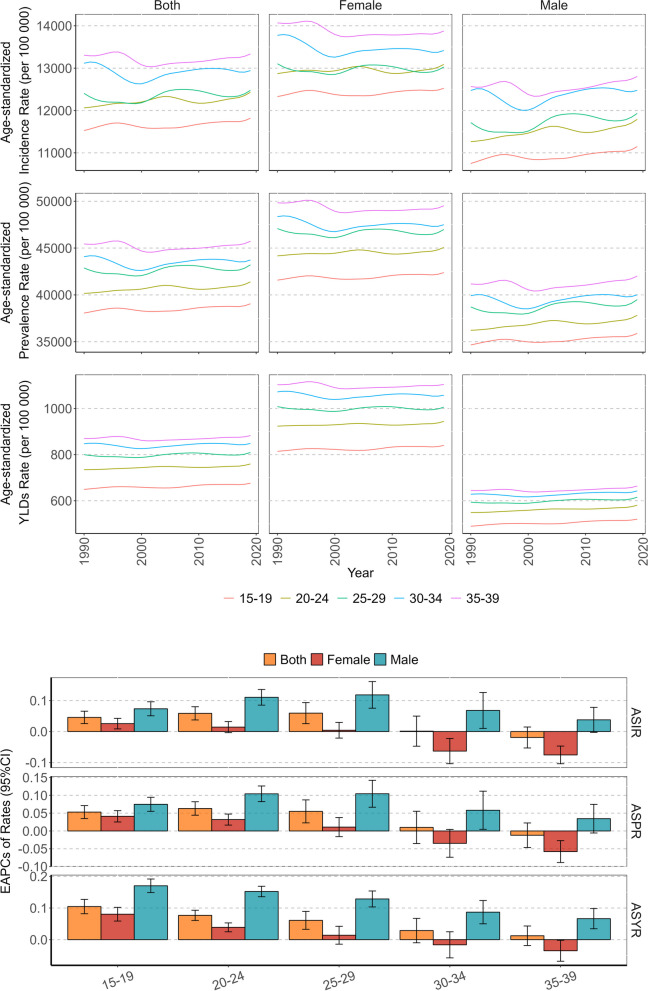
Temporal trend of headache disorders burden in adolescent and young adults by sex and age groups from 1990 to 2019

In 2019, the number of new and prevalent cases of headache in females was approximately 192.98 (95% UI: 161.85–226.34) millions and 677.16 (95% UI: 608.92–746.60) millions, and YLDs caused by headache were 14.47 (95%UI: 2.28–32.90) millions, accounting for 51.69%, 53.64% and 61.52% of all genders, respectively. Additionally, the ASIR, ASPR, and ASYR of females were 13,156.78 (95% CI: 9041.93, 17,651.94) per 100,000, 46,128.79 (95% CI: 38,455.69, 54,311.69) per 100,000, and 984.09 (95% CI: 153.51, 2261.73) per 100,000 (Table 1). The ASRs of females were higher than those of males, especially ASYR, which was 1.6 times higher in females. From 1990 to 2019, in females, there was no statistically significant change in the incidence rate, and the prevalence rate and YLDs rate showed upward trends. The EAPCs of ASIR, ASPR, and ASYR were -0.01 (95% CI: -0.02, 0.01), 0.02 (95% CI: 0.01, 0.03), and 0.04 (95% CI: 0.03, 0.06), respectively. In males, the incidence rate, prevalence rate, and YLDs rate exhibited rising trends. The EAPCs of ASIR, ASPR, and ASYR were 0.09 (95% CI: 0.08, 0.11), 0.09 (95% CI: 0.07, 0.11), and 0.14 (95% CI: 0.12, 0.16), respectively, which were more pronounced than those in females (Table 1, Figs. 1, 2 and 6).

In the five age groups, this burden exhibited an increasing trend with age. Individuals aged 35–39 had the highest burden, with an ASIR, ASPR and ASYR of 13,335.42 (UI: 9028.63, 17,808.10) per 100,000, 45,733.50 (95% UI: 38,632.83, 53,603.47) per 100,000, and 882.38 (95% UI: 174.08, 1900.34) per 100,000, respectively. However, in terms of the trend, the incidence, prevalence, and YLDs rate in the younger age groups tended to increase, especially among males. The EAPCs of ASIR, ASPR, and ASYR in males aged 20–24 were 0.11 (95% CI: 0.08, 0.14), 0.10 (95% CI: 0.08, 0.13), and 0.15 (95% CI: 0.13, 0.17), respectively. In the older age group of females, this burden exhibited a declining trend. The EAPCs of ASIR, ASPR, and ASYR in females aged 35–39 were -0.08 (95% CI:-0.10, -0.05), -0.06 (95% CI: -0.09, -0.03), and -0.04 (95% CI: -0.07, -0.01), respectively (Fig. 6).

### Correlations between the SDI and ASRs

Using scatter plots, the correlation between the SDI and the ASRs of headaches in different countries was depicted (Figure S4). Pearson correlation analysis revealed a positive correlation between SDI and ASRs of headaches individuals aged 15–39 (*ρ*_1_ = 0.556 and *P*_1_ < 0.001, *ρ*_2_ = 0.586 and *P*_2_ < 0.001, *ρ*_3_ = 0.470 and *P*_3_ < 0.001, respectively).

## Discussion

### Principal findings

In this study, we have obtained several conclusive findings: the global burden of headache disorders in AYAs is severe, with a large number of affected individuals and a significant number of YLDs. Furthermore, this burden has been steadily increasing over the past three decades. Additionally, the distribution and trends of this burden show distinct characteristics closely associated with factors such as regional socioeconomic development, age, and gender. Moreover, in the global population aged 15–39 years, migraine has a lower incidence and prevalence compared to TTH, but it leads to significantly higher YLDs than TTH. A comprehensive assessment of the burden of headache disorders in AYAs supplements the existing literature on the burden of headaches in the general population, providing valuable information for global efforts to alleviate the burden of headaches and address health disparities and inequities in headache control strategies.

### Regional difference in the burden of headache disorders in adolescents and young adults

In this study, we found that countries or regions with higher levels of socioeconomic development bear a heavier burden of headache disorders among AYAs, whether it is in terms of incidence, prevalence, or YLDs rate. Effective healthcare plays a crucial role in reducing the burden of headache disorders, but inadequate diagnosis and treatment are common issues in the global headache prevention and management [20]. In countries or regions with medium levels of SDI, the notable trend is an increasing burden. Given the increasing burden of headache disorders among the global population aged 15–39 years revealed in this study, it is important for all countries to enhance headache diagnosis, strengthen their healthcare systems, and allocate resources for treatment. However, it may be necessary for countries with higher levels of socioeconomic development to place additional emphasis on specific aspects of healthcare resource allocation, while countries with lower to moderate levels of economic development may need to prioritize the overall strengthening of their healthcare systems [21].

### Types of characteristics in the burden of headache disorders in adolescents and young adults

According to our study, the incidence and prevalence of TTH in individuals aged 15–39 years are 7.5 times and 1.7 times higher, respectively, than those of migraine. In contrast, migraine causes 11 times more YLDs than TTH, especially among females. This may be related to the intensity of pain and accompanying symptoms associated with migraine [22]. Compared to TTH, migraine is characterized by higher pain severity, leading patients to often require rest or have restricted activity, and experiencing neurological symptoms such as nausea and vomiting [23]. Additionally, the distribution of headache types varies slightly among countries or regions with different SDI levels. This suggests that when developing population-based interventions for headaches in AYAs, it is important to consider the characteristics of headache type distribution in conjunction with factors such as socioeconomic development level, age, and gender.

### Sex difference in the burden of headache disorders in adolescents and young adults

In this study, we found that gender and age are important factors influencing the distribution and trends of headache burden among individuals aged 15–39 years. Compared to males, females in the AYAs group bear a heavier burden of headache disorders. One possible reason is the significant role of physiological factors [24]. Females experience hormonal fluctuations during puberty, particularly in relation to estrogen and progesterone levels during the menstrual cycle, which are closely associated with headache attacks. Furthermore, sex hormones may interact with factors such as nerve conduction, vascular constriction and dilation, and inflammatory responses, contributing to the severity of headaches [25]. On the other hand, females are more susceptible to the impact of psychological and social factors such as stress, emotional changes, irregular sleep patterns, and dietary irregularities, leading to more severe headaches [26]. As for young males, it is worth noting that the burden of headache disorders has exhibited a more significant upward trend over the past three decades.

### Age difference in the burden of headache disorders in adolescents and young adults

In all age groups included in this study, the burden of headache disorders becomes increasingly heavy with age. Mental stress is prevalent in AYAs [27]. Poor sleep quality and insufficient sleep, which can be related to stress and excessive use of electronic devices, serve as triggering factors for headaches in young adults [28]. Additionally, unhealthy lifestyle factors such as lack of exercise, prolonged sedentary behavior, and excessive caffeine intake in their diet, which are commonly found in this age group, may increase the risk of headaches [29, 30]. It's important to note the changing trend of increasing burden of headache disorders in younger individuals, typically around the age of 20. The school environment and family support may play a significant role in the sensitivity of adolescents to external factors and are closely related to their physical health [31]. Therefore, it is recommended to give adequate attention to these factors in headache interventions for AYAs.

### Strengths and limitations of this study

In this study, we utilized the data from GBD 2019 to, for the first time, present the most standardized and comprehensive distribution and trends of headache disorders burden in the global population aged 15–39. We also investigated the regional distribution, age, and gender characteristics of this burden. However, our study had several limitations. Firstly, there is a substantial amount of objectively existing headache cases that cannot be classified according to existing diagnostic criteria. Combined with factors such as the lack of attention and delayed diagnosis of headaches, this burden in AYAs may currently be underestimated. Secondly, in some countries, particularly those with limited healthcare services, there is a lack of capacity to conduct epidemiological research on headaches at the population level. This can result in bias in the estimates of the data in the GBD 2019 study. Thirdly, when utilizing global population rates for age-standardization calculations, the resulting rates come with confidence intervals. This necessitates cautious interpretation of the study findings and underscores the need for additional real-world research to validate the results. Additionally, the confounding effect of improved headache diagnosis and public awareness on the increase in headache burden in AYAs over the last three decades cannot be estimated at this time.

## Conclusions

Headache among the global population aged 15–39 presents a challenging public health task. A comprehensive assessment of the burden of headache disorders in adolescents and young adults supplements the existing literature on the burden of headaches in the general population, providing valuable insights for global efforts to alleviate the burden of headaches and address health disparities and inequities in headache control strategies. Special attention should be given to specific characteristics, such as the higher prevalence of severe headaches in females, and the increasing trend in males and younger age groups. These characteristics call for targeted intervention measures.

### Supplementary Information

**Additional file 1: Figure S1. **Proportion of headache disorders types globally in adolescent and young adults by SDI in 2019.

**Additional file 2: Figure S2. **Proportion of headache disorders types globally in adolescent and young adults by sex in 2019.

**Additional file 3: Figure S3. **Proportion of headache disorders types globally in adolescent and young adults by age groups in 2019.

**Additional file 4: Figure S4. **The correlation between the SDI and the ASRs of headaches in different countries or regions.

## Data Availability

Data used in the analyses can be obtained from the Global Health Data Exchange Global Burden of Disease Results Tool (https://ghdx.healthdata.org/gbd-results-tool).
