# Supplementary figures and images for: The burden and trends of headache disorders among the population aged 15–39: a study from 1990 to 2019
Source: J Headache Pain. 2023 Dec 13;24(1):168. doi: 10.1186/s10194-023-01703-0 (PMC10717103; doi:10.1186/s10194-023-01703-0)

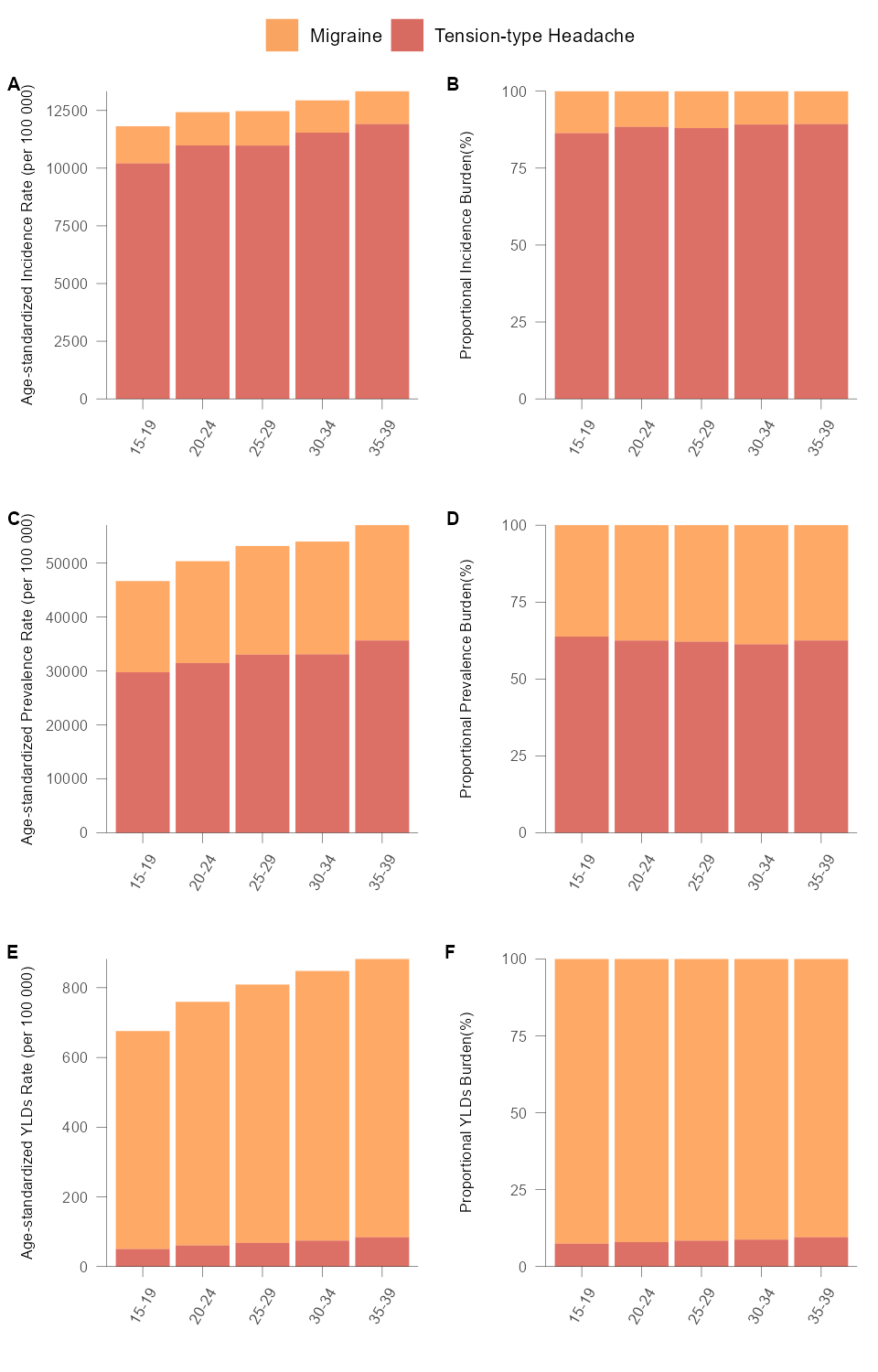

Supplement: Supplementary file 1 — Additional file 1: Figure S1. Proportion of headache disorders types globally in adolescent and young adults by SDI in 2019. [file 10194_2023_1703_MOESM1_ESM.png]

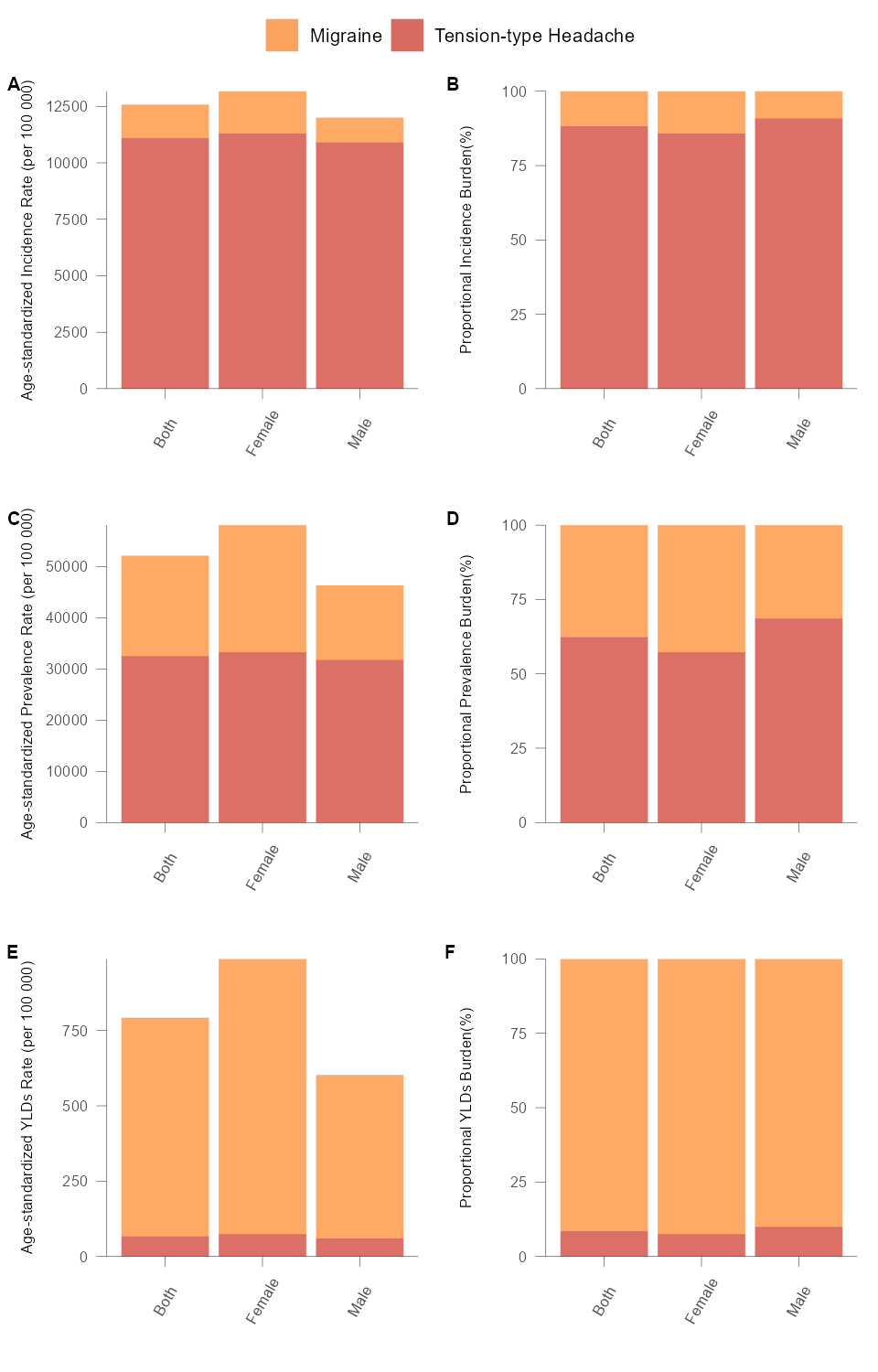

Supplement: Supplementary file 2 — Additional file 2: Figure S2. Proportion of headache disorders types globally in adolescent and young adults by sex in 2019. [file 10194_2023_1703_MOESM2_ESM.png]

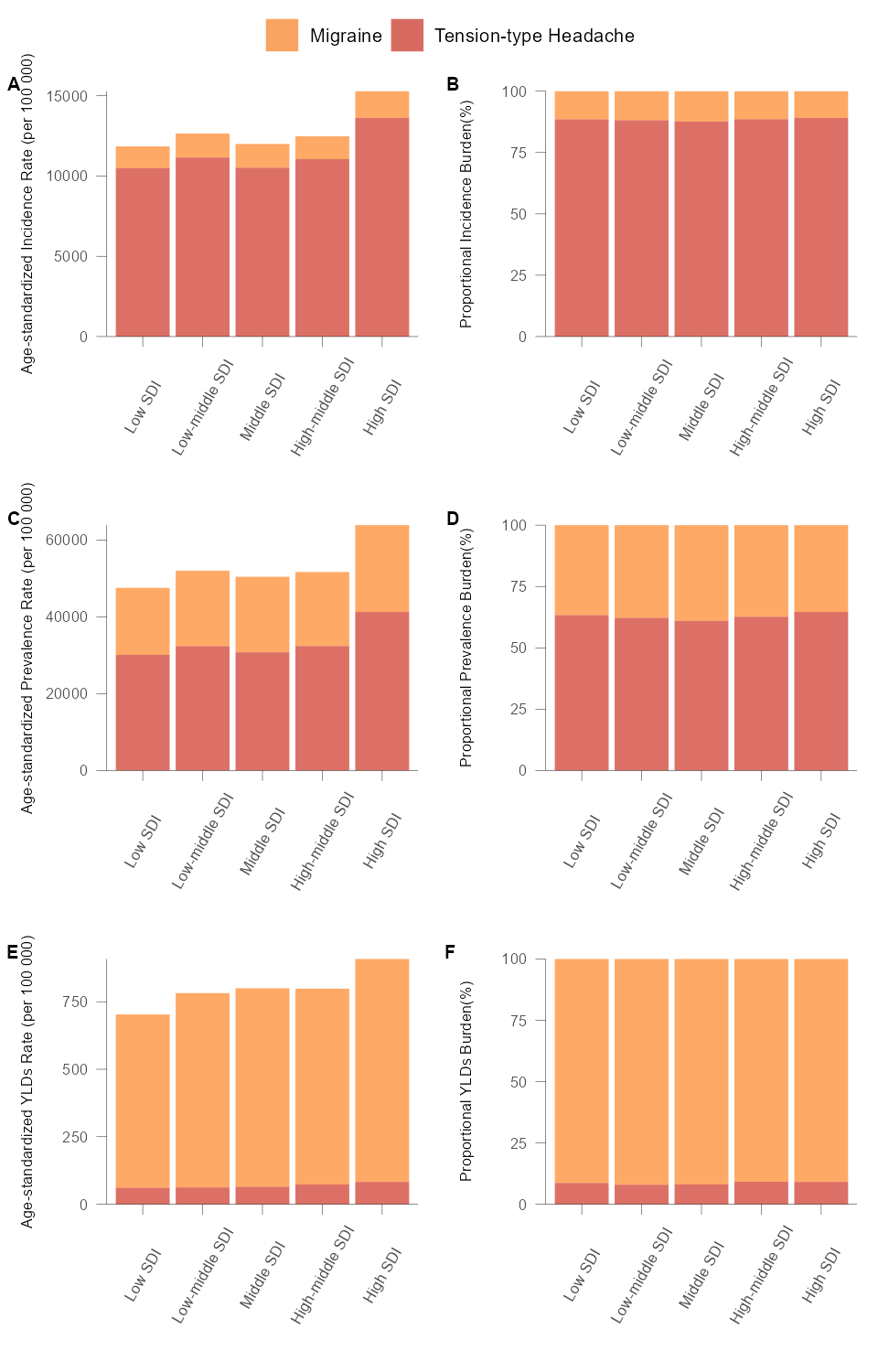

Supplement: Supplementary file 3 — Additional file 3: Figure S3. Proportion of headache disorders types globally in adolescent and young adults by age groups in 2019. [file 10194_2023_1703_MOESM3_ESM.png]

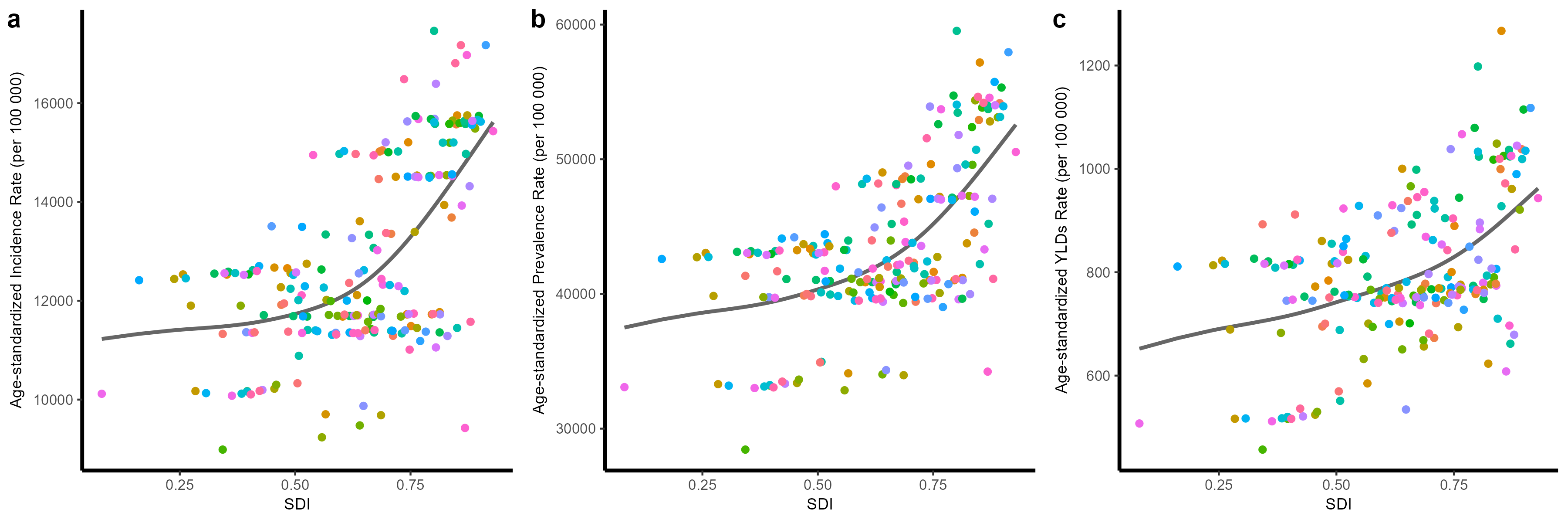

Supplement: Supplementary file 4 — Additional file 4: Figure S4. The correlation between the SDI and the ASRs of headaches in different countries or regions. [file 10194_2023_1703_MOESM4_ESM.png]
